# Impact of Priming Volume Reduction on Hematocrit Retention in Pediatric Cardiopulmonary Bypass: A Retrospective Analysis

**DOI:** 10.1002/pan.70173

**Published:** 2026-03-24

**Authors:** Tokimitsu Hibino, Yusuke Okui, Yoshie Toba, Satoko Kondo, Hiromi Ikegami, Kiyoshi Suzuki, Fumiko Ogura, Hiroshi Masui, Norihisa Kitamoto, Masaaki Koide, Tsutomu Yamazaki

**Affiliations:** ^1^ Department of Social Medical Sciences Graduate School of Medicine, International University of Health and Welfare Tokyo Japan; ^2^ Department of Anesthesiology Seirei Hamamatsu General Hospital Hamamatsu Japan; ^3^ Department of Clinical Engineering Seirei Hamamatsu General Hospital Hamamatsu Japan; ^4^ Department of Clinical Medical Engineering, Faculty of Medical Sciences Fujita Health University Toyoake Japan; ^5^ Department of Cardiovascular Surgery Seirei Hamamatsu General Hospital Hamamatsu Japan

**Keywords:** blood transfusion, cardiopulmonary bypass, hematocrit, pediatric cardiac surgery, retrograde autologous priming, venous antegrade priming

## Abstract

**Background:**

Retrograde autologous priming and venous antegrade priming replace the cardiopulmonary bypass circuit crystalloid with patient blood to mitigate hemodilution. However, their effectiveness in pediatric patients, particularly when analyzed as continuous variables, remains unclear.

**Aims:**

We aimed to evaluate the effects of autologous priming techniques on blood conservation and patient safety during pediatric cardiac surgery.

**Methods:**

This retrospective cohort study included 191 patients (age 0–14 years; weight > 6 kg) who underwent repair of ventricular and/or atrial septal defects. The primary endpoint was the correlation between the priming volume reduction rate (proportion of priming solution replaced) and the hematocrit retention ratio (hematocrit immediately after cardiopulmonary bypass initiation divided by pre‐bypass hematocrit). Secondary outcomes, including transfusion rates, regional cerebral oxygen saturation, and lactate levels, were compared between a retrograde autologous priming group (*n* = 144) and a control group (*n* = 47). All patients underwent venous antegrade priming.

**Results:**

The priming volume reduction rate correlated positively with the hematocrit retention ratio (Spearman's *ρ* = 0.545, *p* < 0.001). Multiple regression confirmed this independent association: a 0.1 increase in the priming volume reduction rate corresponded to a 0.5% absolute increase in hematocrit at bypass initiation. The retrograde autologous priming group had a significantly higher transfusion‐free surgery rate (93.1% vs. 76.6%; relative risk ratio 1.22; *p* = 0.005). Regarding safety and the postoperative course, no significant intergroup differences were found in trends in regional cerebral oxygen saturation (Time × retrograde autologous priming interaction) or in lactate levels. Similarly, intensive care unit and hospital lengths of stay did not differ significantly between groups. Safety analyses suggested no evidence of cerebral perfusion suppression during retrograde autologous priming.

**Conclusion:**

These findings suggest that even partial retrograde autologous priming is effective to mitigate hemodilution and is independently associated with improved hematocrit retention and a significant reduction in transfusion risk after initiation of cardiopulmonary bypass in pediatric patients.

**Trial Registration:**

This study was registered with the UMIN Clinical Trials Registry, Japan, prior to commencement (Trial ID: R000067879)

## 1 Introduction

Retrograde autologous priming (RAP) and venous antegrade priming (VAP) techniques are performed immediately before the initiation of cardiopulmonary bypass (CPB). We collectively defined these techniques as autologous priming (AP). The CPB circuit is filled with a priming solution consisting of an electrolyte preparation solution and a plasma substitute. When CPB is initiated, the patient's blood becomes diluted by the priming solution. RAP reduces hemodilution by retrogradely replacing the priming solution within the CPB circuit with the blood obtained from the arterial cannula [1]. VAP can reduce the amount of priming solution within venous cannulas by evacuating the solution using vacuum‐assisted drainage [2]. The hemodilution‐reducing effect of AP in adult patients is well established in the literature [3, 4, 5]. However, existing pediatric studies, which often exclude infants [6] and group patients based solely on the presence of RAP [4, 5, 6], may oversimplify the true effect of AP. Therefore, we hypothesized that the rate of priming solution reduction achieved by AP would correlate with the degree of hemodilution at the initiation of CPB. This retrospective study aimed to examine this correlation by analyzing AP as a continuous variable to better characterize its effect on hemodilution.

## 2 Methods

This retrospective study followed STROBE guidelines and was approved by the Clinical Research Ethics Review Board of the International University of Health and Welfare (Approval Number: 25‐TA‐215).

### 2.1 The Patients

We analyzed patients aged 0–14 years who underwent intracardiac repair for ventricular and/or atrial septal defects at Seirei Hamamatsu General Hospital (2006–2024). The cohort of 188 Japanese patients and three patients of mixed Japanese‐Brazilian ancestry included only those who were scheduled for a planned preoperative transfusion‐free surgery. Within this transfusion‐free intent‐to‐treat cohort, we analyzed both patients who successfully avoided allogeneic blood products and those who ultimately required a transfusion during or after surgery for clinical indications. Per institutional protocol, patients who weighed < 6 kg were excluded because they required blood priming.

**FIGURE 1:**
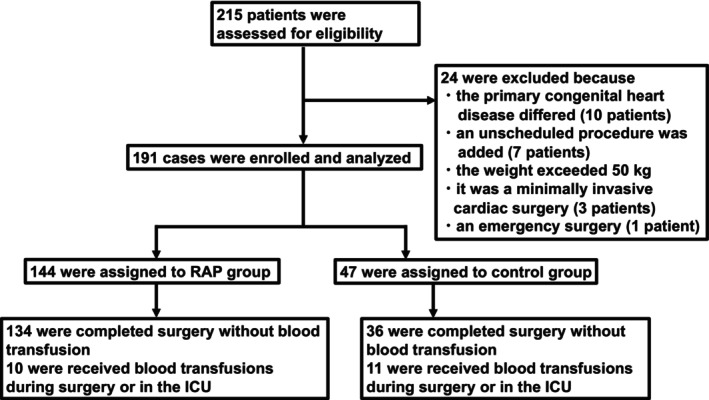
Study flow chart. This diagram illustrates the selection process of the final study population from a cohort of 215 patients who underwent VSD and/or ASD repair at our hospital between 2006 and 2024. A total of 24 patients are excluded based on the exclusion criteria shown in the figure. The remaining 191 patients are divided based on the priming method into the RAP group (*n* = 144), which receives both RAP and VAP, and the control group (*n* = 47), which receives VAP only. A total of 188 patients were included in the primary endpoint analysis; three were excluded because they received blood transfusions immediately after the initiation of cardiopulmonary bypass. For secondary endpoint analyses, cases with missing data are excluded, as appropriate. The number of participants included in each secondary outcome analysis is indicated in the relevant tables and figures. ICU, intensive care unit; RAP, retrograde autologous priming; VAP, venous antegrade priming.

After excluding 24 patients (Figure 1), 191 patients were analyzed (RAP group, *n* = 144; control group, *n* = 47). The cohort included a subgroup of 30 infants (7–11 months) (RAP group, *n* = 21; control group, *n* = 9), representing a population particularly susceptible to hemodilution. In the control group, RAP was omitted because of hypotension (*n* = 30; > 20% baseline decrease) or the need for immediate CPB initiation to manage arterial cannula‐site bleeding (*n* = 17). In these cases, blood loss was recovered and reused via the CPB reservoir.

### 2.2 Outcomes

As the primary endpoint, we defined two primary parameters to evaluate the correlation between AP‐induced priming volume reduction and hemodilution suppression.

- Priming volume reduction rate: Total AP volume divided by the initial priming volume.
- Hematocrit retention ratio: Hematocrit immediately after CPB initiation divided by the pre‐CPB hematocrit.

We then examined the correlation between these two ratios.

Secondary endpoints involved comparison of the RAP and control groups regarding (1) transfusion‐free surgery rate, (2) changes in regional cerebral oxygen saturation (rSO_2_) and lactate levels, and (3) intensive care unit (ICU) and postoperative hospital length of stay (LOS).

### 2.3 Anesthesia and Perioperative Protocol

Standard general anesthesia was maintained using sevoflurane, dexmedetomidine, and opioids. An arterial catheter was inserted into the radial or femoral artery, and a central venous catheter was placed in the femoral vein. After surgery, patients were transferred to the ICU without extubation, and sedation was maintained with dexmedetomidine. The attending anesthesiologist determined the selection and dosing of opioids and inotropes. Blood samples were collected at predefined time points: pre‐surgery; pre‐CPB (3 min after heparin administration); 5 min after CPB initiation; hourly during CPB; at CPB withdrawal; after protamine administration; and at ICU admission.

### 2.4 CPB Protocol and Autologous Priming

CPB circuits were selected based on weight (Table 1) using the COMPO III Neo Heart‐Lung System (Technowood Corporation, Tokyo, Japan). The CPB protocol followed Singab et al. [6] except for an initial heparin dose of 300 units/kg. Following weaning from CPB, modified ultrafiltration was standardly performed in all patients to optimize hematocrit levels and hemodynamic stability.

**TABLE 1:** Cardiopulmonary bypass circuit components and priming solution composition based on body weight.

Patient weight (kg)	Membrane oxygenator	Hard‐shell reservoir	Pump head diameter (mm)	Circuit tube inner diameter (mm) (inch)	Priming volume (mL)
4–7	FX05^a^	RR10^b^	120	6.35 (1/4)	115
7–11	FX05^a^	RR10^b^	150	6.35 (1/4)	125
11–17	FX05^a^	RR10^b^	120	9.53 (3/8)	130
17–30	Quadrox^c^	VHK^d^	150	9.53 (3/8)	230
**Solution**	**Concentration**	**Dose/volume (unit)**			
Mannitol	20%	5 mL/kg			
Bicarbonate	8.4%	1 mL/kg			
Heparin	—	100 units/kg			
Cefazolin	—	30 mg/kg			
HES130000	—	10 mL/kg			
SUBLOOD	—	Remaining volume			

*Note:* The inner diameter of the cardiopulmonary bypass circuit tubes is also commonly expressed in inches; thus, both millimeters and inches are listed.

Abbreviations: HES130000, hydroxyethyl starch 130 000; SUBLOOD, SUBLOOD substitution fluid for hemofiltration BSG (Fuso Pharmaceutical Industries Ltd., Osaka, Japan).

^a^ CAPIOXTM FX05; Terumo Corporation, Tokyo, Japan.

^b^ CAPIOXTM RR10; Terumo Corporation, Tokyo, Japan.

^c^ Quadrox‐iTM HMO31000, Getinge Group, Gothenburg, Sweden.

^d^ VHK31000, Getinge Group, Gothenburg, Sweden.

RAP and VAP were performed per Rosengart et al. [7] RAP was initiated unless systolic blood pressure decreased by > 20% or active bleeding occurred. VAP was performed in all cases. The combination of RAP and VAP aimed to achieve maximal replacement of the priming fluid; although complete replacement remains the theoretical ideal for eliminating hemodilution, volume replacement was maximized in practice within the limits of hemodynamic stability.

### 2.5 Data Extraction

Data were extracted from electronic and handwritten medical records (2006–2024). Items included demographics, anesthetic/surgical variables, rSO2 (NIRO‐200NX, Hamamatsu Photonics Corporation, Hamamatsu, Shizuoka, Japan), lactate, CPB parameters (volume, pressure, and duration), and clinical course.

- CPB Details: CPB circuit priming volume, RAP and VAP volumes, medications and blood products administered during CPB, CPB‐related events, CPB duration, aortic cross‐clamp time, and circuit pressure.

CPB records were handwritten from 2006 to May 2019, whereas records from June 2019 to 2024 were recorded electronically and included minute‐by‐minute vital signs. Because RAP was performed 1–2 min immediately before CPB initiation, rSO_2_ data obtained 2 min before and at the start of CPB were specifically analyzed for the control group.

### 2.6 Statistical Analysis

#### 2.6.1 Data Presentation and Initial Tests

Continuous variables are reported as medians (interquartile range [IQR]) and categorical variables as frequencies. The integrity of preoperative hemoglobin and hematocrit data was verified by graphical assessment, yielding a Spearman's rank correlation coefficient of 0.948.

- Differences in categorical variables were assessed using Fisher's exact test.
- Differences in continuous patient factors (e.g., age and weight) were analyzed using the Mann–Whitney U test due to non‐normal distribution.

The correlation between the primary endpoint variables (priming volume reduction rate and hematocrit retention ratio) was determined using Spearman's rank correlation coefficient.

#### 2.6.2 Multiple Regression Analysis

A multiple regression analysis was performed to examine the relationship between the hematocrit retention ratio (dependent variable) and its explanatory variables. The final model was constructed using backward elimination, retaining variables with *p* < 0.05. The initial model included the following seven variables:

1. Priming volume reduction rate.
2. Four biological and clinical factors (sex, weight, preoperative hematocrit, and immediate pre‐RAP dopamine infusion rate) were included based on previous studies [6, 8].
3. Presence of trisomy 21, which is associated with lower surgical weight [9].
4. Presence of heart failure, which is associated with earlier surgical intervention [10].

The two latter conditions (trisomy 21 and heart failure) were included because they are known to influence patient size and surgical timing, which critically affect hemodilution.

Model assumptions were visually assessed using residuals versus Fitted Values (homoscedasticity), Normal Q‐Q plots (normality), and residuals versus leverage (influential outliers). Multicollinearity was assessed using the variance inflation factor (VIF), with a VIF < 10 as the acceptable limit. The goodness‐of‐fit of the model was evaluated using the adjusted coefficient of determination (adjusted R^2^) and the F‐statistic. The effects of individual variables were reported using the regression coefficient (*β*), standard error, 95% confidence interval (CI), and *p*‐value.

#### 2.6.3 Secondary Endpoints

Secondary endpoints were analyzed as follows:

- Transfusion‐free rates were compared using relative risk (RR).
- rSO_2_ and lactate trends were analyzed using Aligned Rank Transform ANOVA (ART‐ANOVA) to evaluate Time × RAP interactions, providing robustness against imbalanced sample sizes and non‐normal distributions.

Blood Pressure Check: Baseline blood pressure (pre‐RAP/CPB 2 min) was higher in the RAP group; however, the Mann–Whitney *U* test showed no significant intergroup difference at CPB initiation. Within‐group blood pressure changes were assessed using the Wilcoxon signed‐rank test.

rSO_2_ Analysis Strategy: rSO_2_ changes were initially assessed using Wilcoxon's signed‐rank test for within‐group comparisons. Given the disparity in sample sizes (e.g., right side rSO_2_; RAP *n* = 131 vs. control *n* = 44), ART‐ANOVA was subsequently used to robustly evaluate the Time × RAP interaction effect (i.e., difference in change patterns).

Multiple Comparisons: Bonferroni adjustment was applied to the Mann–Whitney *U* tests and Wilcoxon's signed‐rank tests, but not to the F‐test for the Time × RAP interaction in the ART‐ANOVA, as this analysis evaluates a single fixed effect.

Lactate Levels: Using an ART‐ANOVA, changes across five of the time points described in the anesthesia protocol were examined: before CPB (3 min after heparin administration), 5 min after CPB initiation, at CPB withdrawal, after protamine administration, and at ICU admission.

Length of Stay: ICU and postoperative hospital LOS were dichotomized and analyzed using the Mann–Whitney *U* test.

#### 2.6.4 Subgroup Analysis

Given the potential physiological differences across age groups, a subgroup analysis was performed for infants (*n* = 30) (7–11 months) to evaluate the consistency of the findings. The Spearman's rank correlation coefficient was similarly applied to this cohort.

#### 2.6.5 Software and Significance

Patients with missing data were excluded. All statistical analyses were performed using EZR version 1.68 (Jichi Medical University), which is a graphical user interface for R version 4.3.1 (The R Foundation for Statistical Computing, Vienna, Austria) [11]. The level of statistical significance was set at *p* < 0.05 for two‐tailed tests.

## 3 Results

### 3.1 Baseline Characteristics

Baseline characteristics, anesthetic, and surgical factors were comparable between the RAP (*n* = 144) and control (*n* = 47) groups (Table 2).

**TABLE 2:** Baseline patient characteristics according to treatment assignment.

Characteristic	RAP (*n* = 144)	Control (*n* = 47)	*p*
Age Mean (month)	38.5 (17–87)	28.0 (13–75)	0.221
Sex Male/Female	79/65	23/24	0.504
Height (cm)	92.6 (76–117.6)	83.3 (73–115.4)	0.188
Weight (kg)	13.6 (9.4–21.2)	11.4 (7.8–18.6)	0.164
Birthweight (g)	2925 (2575–3199)	2910 (2595–3419)	0.508
CHD (VSD/ASD/both)	78/59/7	32/14/1	0.278
RCCP (yes/no)	38/106	13/34	0.852
DCRV (yes/no)	1/143	0/47	1.00
Heart failure (yes/no)	56/88	22/25	0.394
21 trisomy (yes/no)	12/132	5/42	0.571
Pulmonary hypertension (yes/no)	33/111	16/31	0.177
Pulmonary artery banding (yes/no)	7/137	5/42	0.173
PDA (no/untreated/ligation/occluder)	136/3/4/1	44/2/1/0	0.879
Pulmonary stenosis (yes/no)	127/17	42/5	1.00
Anesthesia (fentanyl/remifentanil)	20/124	11/36	0.170
Anesthesia time (min)	312 (281–338.3)	315 (284.5–337.5)	0.851
Operation time (min)	238 (205–262)	241 (215.5–264)	0.565
Aortic cross clamp time (min)	48.0 (36.0–63.0)	50 (38.5–70.0)	0.356
Infusion volume (mL)	385 (274.5–621)	325 (248–538.5)	0.244
Transfusion of residual pump blood (mL)	43.0 (35.8–56.3)	46.0 (35.5–55.0)	1.00
Blood loss (mL)	27.6 (15.4–50.1)	27.5 (17.0–83.5)	0.409
Urine volume (mL)	370.5 (201.8–548)	296 (166.5–509.5)	0.391

*Note:* Categorical variables are assessed using Fisher's exact test, and continuous and non‐normally distributed variables are assessed using the Mann–Whitney *U* test. No significant differences in baseline patient characteristics are observed between the two groups.

Abbreviations: ASD, atrial septum defect; CHD, congenital heart disease; DCRV, double‐chambered right ventricle; PDA, patent ductus arteriosus; RAP, retrograde autologous priming; RCCP, right coronary cusp prolapse; VSD, ventricular septal defect.

### 3.2 Intraoperative Priming and Hematocrit Changes

Table 3 summarizes the priming profiles and hematocrit levels. In the overall population, although the initial priming volumes were similar between groups, the RAP group achieved a significantly higher AP volume (71.5 vs. 25.0 mL, *p* = 1.53 × 10^−14^) and priming volume reduction rate (0.46 vs. 0.18, *p* = 2.15 × 10^−21^) than the control group. Consequently, the RAP group maintained a higher hematocrit immediately post‐CPB initiation (26.7 vs. 24.8%, *p* = 0.00720) and required fewer blood transfusions (6.9% vs. 23.4%, *p* = 0.00519). In the infant subgroup, although the priming volume reduction rate remained significantly higher in the RAP group (*p* = 0.000720), no significant differences were observed in post‐initiation hematocrit (*p* = 1.00) or transfusion rates (*p* = 0.195).

**TABLE 3:** Intraoperative priming profiles and hematocrit changes: overall population and infant subgroup.

Overall	RAP (*n* = 144)	Control (*n* = 47)	*p*
Priming management			
Initial priming volume (mL)	140.0 (135.0–250.0)	145.0 (138.5–240.0)	1.00^a^
AP volume (mL)	71.5 (55.8–118.5)	25.0 (21.0–37.5)	1.53 × 10^−14^ ^a^
Priming volume reduction rate	0.46 (0.39–0.56)	0.18 (0.12–0.21)	2.15 × 10^−21^ ^a^
Hematocrit (%)			
Pre‐CPB	31.5 (29.7–33.3)	31.2 (29.0–32.3)	1.00^a^
Post‐initiation of CPB	26.7 (24.6–29.3)	24.8 (23.3–32.3)^c^	0.00720^a^
Allogeneic blood transfusion, *n* (%)	10 (6.9%)	11 (23.4%)	0.00519^b^
**Infant subgroup**	**RAP (*n* = 21)**	**Control (*n* = 9)**	***p***
Priming management			
Initial priming volume (mL)	130.0 (115.5–135.0)	145.0 (126.2–145.0)	0.855^a^
AP volume (mL)	48.0 (33.0–67.0)	27.0 (22.0–28.0)	0.00515^a^
Priming volume reduction rate	0.37 (0.29–0.48)	0.19 (0.17–0.21)	0.000720^a^
Hematocrit (%)			
Pre‐CPB	28.8 (26.4–30.3)	28.2 (26.6–30.3)	1.00^a^
Post‐initiation of CPB	22.6 (21.8–23.5)	22.1 (21.3–24.2)^d^	1.00^a^
Allogeneic blood transfusion, *n* (%)	4 (19.0%)	4 (44.4%)	0.195^b^

Abbreviations: AP, autologous priming; CPB, cardiopulmonary bypass; RAP, retrograde autologous priming.

^a^ Mann–Whitney *U* tests for between‐group comparison with Bonferroni adjustment for multiple comparisons.

^b^ Fisher's exact test (nominal *p*‐value).

^c^ Two cases are excluded due to blood transfusion immediately following the initiation of CPB.

^d^ One case is excluded due to blood transfusion immediately following the initiation of CPB.

### 3.3 Main Findings: Efficacy of AP on Hematocrit Retention

#### 3.3.1 Correlation With Priming Volume Reduction Rate

**FIGURE 2:**
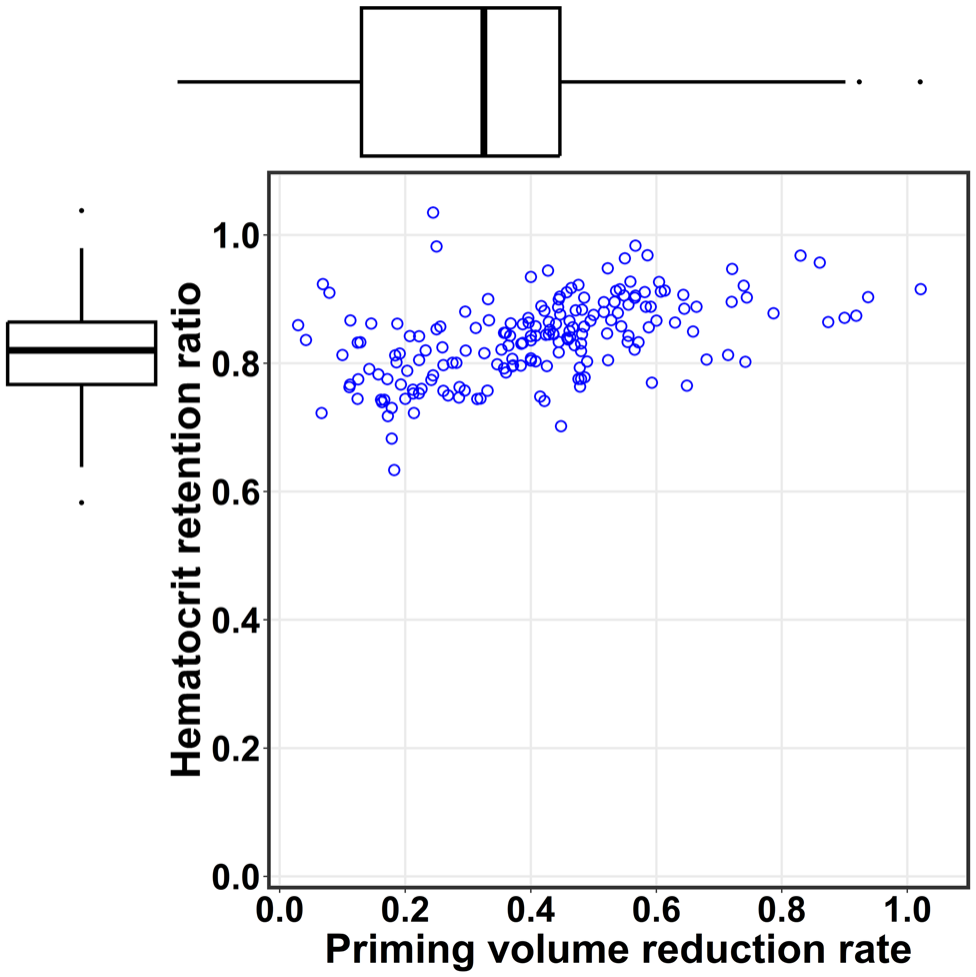
Effect of reducing hemodilution by reduction of CPB priming. This scatter plot illustrates the relationship between the priming volume reduction rate (*x*‐axis) and hematocrit retention ratio (*y*‐axis). Each data point represents a single patient. A significant positive correlation is observed between these two variables (Spearman's correlation coefficient, 0.545; 95% CI, 0.427–0.652; *p* = 6.45 × 10^−16^). CI, confidence interval; CPB, cardiopulmonary bypass.

Spearman's rank correlation (Figure 2) showed a significant positive correlation between the reduction rate and Hematocrit retention ratio (*ρ* = 0.545; 95% CI, 0.427–0.652; *p* = 6.45 × 10^−16^). Three patients requiring immediate transfusion at CPB initiation were excluded to specifically assess the correlation between reduced hemodilution at CPB initiation and the initial priming volume reduction achieved by AP.

#### 3.3.2 Multiple Regression Analysis

Model diagnostics confirmed robustness against multicollinearity (VIF < 1.629), homogeneity of variance, and the absence of influential outliers (Cook's distance < 0.5). The large sample size (*n* = 188), based on the Central Limit Theorem, ensured the robustness of the coefficients despite minor deviations from normality.

The overall model was highly significant (*F* (5, 182) = 26.86, *p* = 2.20 × 10^−16^), with an adjusted *R* ^2^ of 0.409, explaining 40.9% of the variation in the hematocrit retention ratio. Among five significant predictors, the priming volume reduction rate had the strongest positive association (*β* = 0.129, 1.10 × 10^−9^), while trisomy 21 had the strongest negative association (Table 4). This correlation remained robust in the infant subgroup (*ρ* = 0.625; 95% CI, 0.336–0.807; *p* = 0.000286).

**TABLE 4:** Outcomes: Multiple regression analysis for hematocrit retention ratio (final model).

Variables	VIF	*β*	SE	95% CI	*t*	*p*
Intercept	—	0.790	0.0144	0.761, 0.818	54.9	3.43 × 10^−115^
Priming volume reduction rate	1.13	0.129	0.0200	0.0892, 0.168	6.43	1.10 × 10^−9^
21 trisomy	1.18	−0.0355	0.0136	−0.0624, −0.00872	−2.61	0.00968
Weight	1.62	0.00104	0.000459	0.000134, 0.00194	2.27	0.0246
Heart failure	1.63	−0.0209	0.00935	−0.0393, −0.00242	−2.23	0.0268
Pre dopamine dose (μg/kg/min)	1.27	−0.00564	0.00265	−0.0109, −0.000409	−2.13	0.0347
Pre hematocrit (%)^†^	—	—	—	—	—	—
Sex^†^	—	—	—	—	—	—
	**Value**					
Adjusted *R*‐squared	0.409					
*F* (5, 182)	26.9					2.20 × 10^−16^
Sample size	188					

*Note:* The priming volume reduction rate shows the strongest positive effect and trisomy 21 shows the strongest negative effect. Variables marked with † are excluded from the model using the backward elimination method, and their regression coefficients are therefore not shown in the final model. The *p*‐values are ordered within the variables that are significant at *p* < 0.05. VIF values are below the commonly accepted threshold of 10, indicating no serious multicollinearity issues.

Abbreviations: *β*, regression coefficient; CI, confidence interval; SE, standard error; VIF, variance inflation factor.

### 3.4 Secondary Findings: Transfusion Rate, Safety, and Postoperative Course

#### 3.4.1 Transfusion Rate Comparison (RAP vs. Control)

The RAP group demonstrated a significantly higher transfusion‐free surgery rate (93.1% vs. 76.6%; RR 1.21, 95% CI 1.03–1.43; *p* = 0.00519; Figure 1, Table 3).

#### 3.4.2 Effects of RAP on Cerebral Circulation

**FIGURE 3:**
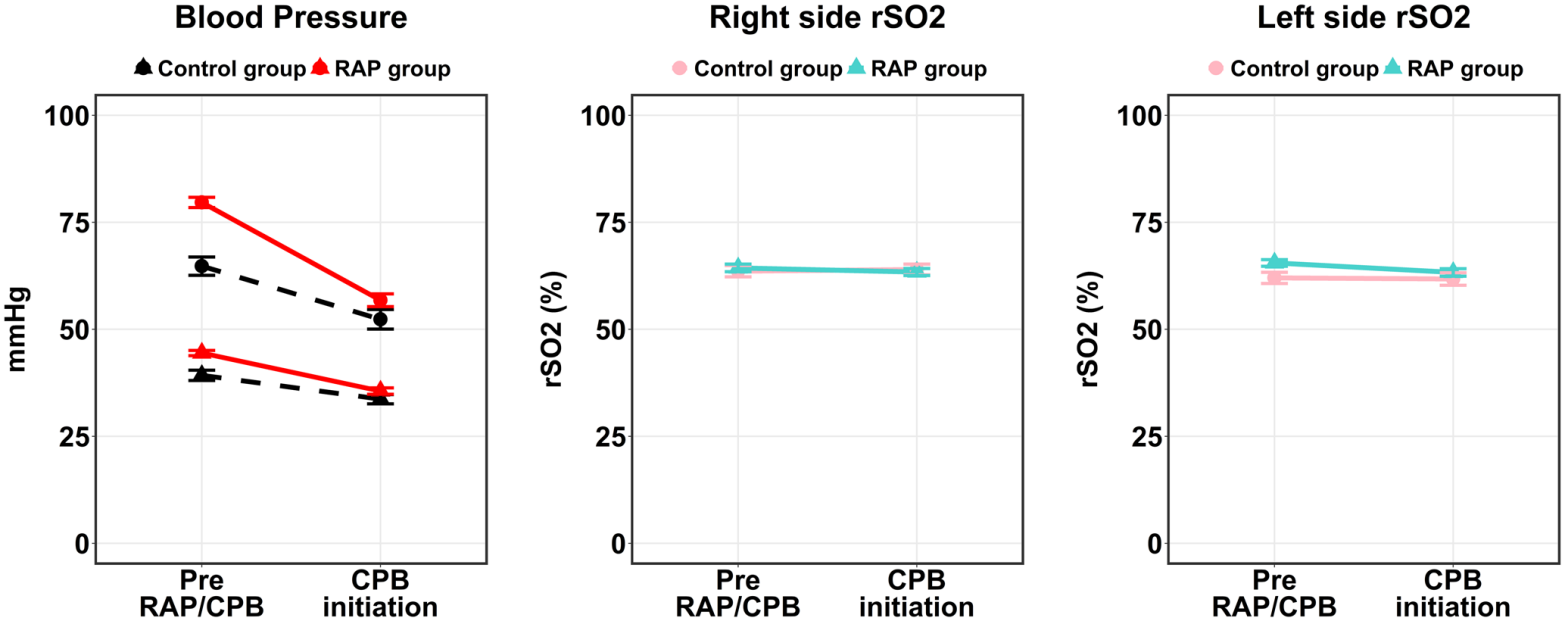
Temporal changes in blood pressure and rSO_2_ before and after initiation of cardiopulmonary bypass. Changes in blood pressure (systolic and diastolic) and right and left rSO_2_ values over time are shown. Blood pressure decreases significantly in both groups following procedure/CPB initiation, but no significant difference in post‐CPB initiation blood pressure is observed between groups. For right and left rSO_2_, a significant decrease over time (main effect of time) is observed, but no significant interaction between group and time is observed. BP, blood pressure; CPB, cardiopulmonary bypass; RAP, retrograde autologous priming; rSO_2_, regional cerebral oxygen saturation.

Regarding cerebral safety, baseline blood pressure was higher in the RAP group, but no significant intergroup difference existed at CPB initiation (Table 5). While within‐group rSO_2_ declined in the RAP group (Right: *p* = 0.00637, Left: *p* = 0.00812), ART‐ANOVA identified no significant Time × RAP interaction effect for either hemisphere (right rSO_2_: *F* (1, 168) = 1.70, *p* = 0.194; left rSO_2_: *F* (1, 166) = 0.948, *p* = 0.332), indicating consistent rSO_2_ change patterns between groups (Figure 3).

**TABLE 5:** Outcomes: Changes in blood pressure and regional cerebral oxygen saturation (rSO_2_) during RAP.

		RAP *n* = 143	Control *n* = 47	RAP vs. control *p*_adj_
Pre RAP/CPB systolic BP (mmHg)		80 (70; 90.5)	62 (54; 73)	2.20 × 10^−7^ ^a^
CPB initiation systolic BP (mmHg)		58 (44.5; 68.5)	52 (43.5; 60.5)	1^a^
Pre‐post comparison *p*_adj_		4.70 × 10^−21^	2.11 × 10^−4^	
Pre RAP/CPB diastolic BP (mmHg)		43 (39; 49)	40 (32.5; 45)	0.0145^a^
CPB initiation diastolic BP (mmHg)		36 (31; 41)	33 (29; 39)	1^a^
Pre‐post comparison *p*_adj_		5.15 × 10^−18^ ^b^	0.00542^b^	
		**RAP *n* = 131**	**Control *n* = 44**	**RAP vs. control**
Right side rSO_2_	Pre RAP/PreCPB	66 (60; 71)	65 (57.8; 70.3)	1^a^
	CPB initiation	63.8 (57.3; 70.0)	64.5 (59; 70.5)	1^a^
	Difference	−1.4 (−4.48; 1.0)	−0.15 (−3; 3.33)	1^a^
	Pre‐post comparison	0.00637^b^	1^b^	
		**RAP *n* = 130**	**Control *n* = 42**	**RAP vs. control**
Left side rSO_2_	Pre RAP/PreCPB	66 (60; 71.5)	61.5 (57; 69.2)	0.626^a^
	CPB initiation	64.0 (57.3; 70.0)	62.5 (54.3; 69.3)	1^a^
	Difference	−2.0 (−5; 2.0)	−0.5 (−4; 2.88)	1^a^
	Pre‐post comparison	0.00812^b^	1^b^	
ART‐ANOVA		**Right side Pre‐post rSO**_**2**_	**Left side Pre‐post rSO**_**2**_	
Time factor	*F* (df_effect_, df_error_)	6.87 (1, 168)	11.0 (1, 166)	
	*p*	0.00959^c^	0.00107^c^	
Group factor	*F* (df_effect_, df_error_)	0.174 (1, 168)	3.02 (1, 166)	
	*p*	0.678^c^	0.0843^c^	
Interaction	*F* (df_effect_, df_error_)	1.70 (1, 168)	0.948 (1, 166)	
	*p*	0.194^c^	0.332^c^	

*Note:* Data are presented as medians and interquartile ranges. The “Pre” measurement refers to the time before RAP in the RAP group and 2 min before CPB initiation in the control group. Systolic and diastolic BP values are significantly higher in the RAP group at baseline; however, BP at CPB initiation shows no significant intergroup differences. Both groups experience a significant post‐procedural decrease in BP. The bilateral rSO_2_ also shows a significant decline in the RAP group upon within‐group comparison, whereas no significant changes are observed in the control group. The ART‐ANOVA for rSO_2_ reveals a significant overall decline (time main effect), but the Time × RAP interaction effect, which evaluates the difference in the pattern of change between the groups, is not significant.

Abbreviations: ART‐ANOVA, aligned rank transform analysis of variance; BP, blood pressure; CPB, cardiopulmonary bypass; df_effect_, degrees of freedom for the effect; df_error_, degrees of freedom for the error; *p*_adj_, adjusted *p*‐value; RAP, retrograde autologous priming; rSO_2_, regional cerebral oxygen saturation.

^a^ Mann–Whitney *U* tests for between‐group comparison with Bonferroni adjustment for multiple comparisons.

^b^ Wilcoxon's signed rank tests for within‐group comparison with Bonferroni adjustment for multiple comparisons.

^c^ ART‐ANOVA (Time × RAP interaction effect). No adjustments for multiple comparisons are applied to the *F*‐test for the interaction effect because it evaluates a single fixed effect within the model.

Lactate trends similarly showed no significant interaction.

#### 3.4.3 Postoperative Course

No significant difference in ICU LOS or postoperative hospital stay was observed between the RAP and control groups. The 1‐year postoperative survival rate was 100% across all patients.

## 4 Discussion

### 4.1 Correlation With Priming Volume Reduction Rate

Despite increasing recommendations for RAP in pediatric cardiac surgery involving CPB [12, 13, 14], its direct effect on reducing hemodilution remains unclear, since multiple factors influence surgical outcomes. We observed a positive correlation between the priming volume reduction rate and the hematocrit retention ratio (*ρ* = 0.545; 95% CI, 0.419–0.645). As the priming volume reduction rate increased with greater AP application, the hematocrit retention ratio also increased. Furthermore, multiple regression analysis confirmed that the priming volume reduction rate had the strongest positive effect on hematocrit maintenance (*β* = 0.129, *p* = 1.10 × 10^−9^), supporting our hypothesis that greater AP application directly mitigates hemodilution.

### 4.2 Clinical Implications

In this subsection, we translate the abstract ratios used in our primary analysis into absolute values to understand better the practical impact of AP on the initiation of CPB. Based on our regression model, we will now detail how changes in the priming volume reduction rate specifically affect hematocrit levels at the onset of CPB. According to our multiple regression analysis, the *β* coefficient for the priming volume reduction rate was 0.129, indicating that a 0.1 increase in the reduction rate was associated with a 0.0129 increase in the hematocrit retention ratio. To translate this into a clinical context, we can consider a patient with a pre‐CPB hematocrit of 40% and a baseline hematocrit retention ratio of 0.80 without AP. In this scenario, the hematocrit at the initiation of CPB would be 32.0%. If the priming volume reduction rate is increased to 0.1 through AP, the hematocrit retention ratio rises to 0.8129, resulting in a post‐initiation hematocrit of 32.5%. While an absolute increase of 0.5% in hematocrit per 0.1 reduction rate might initially appear modest, the cumulative effect is substantial. In our study, a median priming volume reduction rate of 0.46 corresponds to an estimated 2.3% absolute increase in hematocrit at CPB initiation. This finding is consistent with the results shown in the randomized controlled trial (RCT) by Singab et al. [6], who reported that the RAP group maintained a 2.3% higher hematocrit at CPB initiation than the control group, resulting in a significantly lower transfusion rate (4% vs. 14%). Thus, the 0.5% hematocrit elevation per 0.1 priming volume reduction rate represents a clinically meaningful improvement in blood conservation. This clinical benefit extends to the most vulnerable patients. In our infant subgroup (aged 7–11 months), the median priming volume reduction rate in the RAP group was 0.37, corresponding to an estimated 1.85% increase in hematocrit at CPB initiation. Despite a smaller sample size, the observed transfusion rates (19.0% vs. 44.4%) (Table 3) and robust correlation (*ρ* = 0.625) suggest that AP's physiological benefit is preserved in small‐bodied patients.

Furthermore, despite the wide age and weight range (0–14 years; 6.2–57.4 kg) in our cohort, multiple regression analysis revealed that body weight was not a significant predictor of the hematocrit retention ratio (*β* = 0.00104, *p* = 0.0246). This suggests that the priming volume reduction rate is a more dominant determinant of hemodilution than the absolute size of the patient. While smaller infants generally have less physiological reserve, the use of weight‐adjusted circuits (Table 1) and the relative success of the priming reduction itself appear to mitigate the impact of body size. This finding is particularly encouraging because it suggests that a high AP reduction rate can effectively maintain hematocrit levels regardless of body size, provided the patient meets the minimum weight threshold for the transfusion‐free protocol. Furthermore, our approach of treating AP volume as a continuous variable—rather than a categorical one—reveals that even partial utilization of AP significantly contributes to maintaining hematocrit levels, providing a flexible yet effective strategy for blood conservation in pediatric cardiac surgery.

### 4.3 Effects of RAP on Cerebral Circulation

Concerns persist regarding RAP‐induced hypotension and cerebral perfusion. We examined the rSO_2_ fluctuations before and after RAP. The Wilcoxon signed‐rank test revealed a significant decrease in rSO_2_ in the RAP group (right, *p* = 0.00637; left, *p* = 0.00812). In contrast, ART‐ANOVA identified no significant Time × RAP interaction (right: *F* (1, 168) = 1.70, *p* = 0.194; left: *F* (1, 166) = 0.948, *p* = 0.332; Table 5). This discrepancy underscores the limitations of simple rank‐based comparisons in factorial study designs. We adopted the ART‐ANOVA approach, which is widely regarded as a robust standard for evaluating interaction effects in nonparametric factorial designs, where traditional rank‐based methods are often unsuitable [15, 16]. The larger sample size of the RAP group in the Wilcoxon test (right side: RAP *n* = 131 vs. control, *n* = 44 cases) conferred greater statistical power to that group. ART‐ANOVA minimizes the impact of outliers and skewed distributions. Therefore, the ART‐ANOVA evaluation of the Time × RAP interaction is less susceptible to non‐normality, heteroscedasticity, and imbalanced sample sizes. Based on the robust ART‐ANOVA analysis, we concluded that the pattern of rSO_2_ fluctuation (Time × RAP interaction) did not differ significantly between the two groups, consistent with the findings of Hodge et al. [17] Additionally, no significant intergroup differences were observed in perioperative lactate levels. In summary, statistical analyses of both rSO_2_ trends and perioperative lactate levels indicated that RAP had no significant effect on cerebral oxygenation or overall perfusion status. However, the control group's composition introduces selection bias, because it primarily consisted of patients who are ineligible for RAP owing to baseline hemodynamic instability. This “high‐risk” profile may mask the comparative effects of RAP. Nonetheless, a previous study in adults by Joshi et al. [18], acknowledging the potential offsetting effects of bleeding in the control group and hypotension in the RAP group, suggested a wide range for the lower limit of cerebral autoregulation (66 mmHg; 95% prediction interval, 43–90 mmHg). Although direct extrapolation to the pediatric population is difficult, this finding suggests that RAP‐induced hypotension may not necessarily lead to cerebral impairment.

### 4.4 Postoperative Course

Consistent with existing pediatric RCTs [6] and adult meta‐analyses [14], RAP significantly increased transfusion‐free surgery rates but did not impact “hard” endpoints like ICU stay. Pediatric patients are more susceptible to priming dilution because of their lower circulating blood volume [19]; therefore, the effect of priming reduction in children may be more pronounced than that in adults. Therefore, an RCT with a larger, patient‐matched sample size is necessary to definitively evaluate the effects of AP.

### 4.5 Limitations

Although these findings are derived from a retrospective cohort study, causal inference is limited, and the potential influence of unmeasured confounders cannot be excluded.

#### 4.5.1 Methodological and Data Limitations

- Blood Sampling Timing Variation: The timing of blood sampling pre‐CPB initiation varied by several minutes as a result of operator‐dependent procedural steps. This variation may have weakened the observed correlation.
- Data Source Heterogeneity: Data records spanning 2006 to 2024, utilizing both handwritten and electronic records, which may have introduced minor inconsistencies in data capturing.
- Generalizability: A potential selection bias exists because patients weighing less than 6 kg were excluded per our institutional protocol requiring blood priming for safety. Therefore, these findings may not be directly generalizable to neonates or extremely small infants.

#### 4.5.2 Non‐Randomized Design (Secondary Endpoints)

Our primary outcomes (continuous data, *n* = 188) remain statistically robust. However, the non‐randomized design presents a significant limitation for the secondary endpoints analyzed in the intergroup comparison (RAP vs. Control). The control group was retrospectively formed by patients in whom RAP was aborted due to intraoperative complications such as hypotension or surgical field bleeding. This introduces a critical selection bias, as the control group inherently represents a population with greater physiological vulnerability or hemodynamic instability than the RAP group. Such pre‐existing physiological differences may confound comparisons of outcomes such as rSO_2_ and lactate levels. Consequently, while our statistical models (e.g., ART‐ANOVA) attempted to mitigate these imbalances, the secondary endpoint results should be interpreted as hypothesis‐generating rather than definitive evidence.

## 5 Conclusion

Complete replacement of the priming solution with AP is challenging in infants and young children because of their low circulating blood volume. However, this study suggests that even partial application of AP may be an effective strategy for mitigating hemodilution after CPB initiation in pediatric patients. The key findings demonstrate a significant positive correlation between the priming volume reduction rate and hematocrit retention ratio. Notably, reducing the priming volume by 10% using AP resulted in a significant and clinically relevant maintenance of hematocrit levels, increasing the post‐CPB initiation hematocrit by at least 0.5%. This finding confirms that AP results in a clinically significant increase in hematocrit retention, even when administered as a fraction of the total priming volume.

AP may reduce transfusion needs without evidence of cerebral circulation suppression; however, larger studies are warranted. Although pediatric cardiac surgery involves fewer cases than adult surgery, this population offers a unique advantage for analyzing hemodilution. Despite their broad physiological spectrum, pediatric patients are typically less affected by the chronic, lifestyle‐related diseases or age‐associated degenerative changes that are frequently present in adults. This relative absence of such confounding factors allows for a more focused assessment of the correlation between AP efficiency and blood conservation. RCTs with matched patients can provide high‐quality evidence. Establishing a standard AP protocol is beneficial for enhancing patient safety and optimizing outcomes in vulnerable populations.

## Funding

The authors have nothing to report.

## Ethics Statement

This study was approved by the Institutional Review Board (IRB) of International University of Health and Welfare, Tokyo, Japan (Approval No. 25‐TA‐215).

## Consent

Given the retrospective nature of this study and the use of anonymized data, the requirement for individual patient‐informed consent was formally waived by the IRB of the International University of Health and Welfare. However, all participants were allowed to opt out of the study through public notice, in accordance with the Ethical Guidelines for Medical and Health Research Involving Human Subjects in Japan.

## Conflicts of Interest

The authors declare no conflicts of interest.

## Data Availability

The data that support the findings of this study are available on request from the corresponding author. The data are not publicly available due to privacy or ethical restrictions.
